# A Dual-Clock Stability Feature-Based Noise-Adaptive Clock Steering Approach for Single-Satellite Time Reference Generation

**DOI:** 10.3390/s26185699

**Published:** 2026-09-08

**Authors:** Yixin Xiang, Lin Chen, Yuqi Liu, Bowen Jiang, Li Li

**Affiliations:** The 29th Research Institute of China Electronics Technology Group Corporation, Chengdu 610036, China; xiangyixinuestc@163.com (Y.X.); liu.yuqi@139.com (Y.L.);

**Keywords:** clock steering, control, Kalman filter, adaptive filter, time reference

## Abstract

**Highlights:**

**What are the main findings?**

- The proposed clock steering method integrating the variational Bayesian adaptive Kalman filter and PID control combines the excellent short-term stability of OCXO and the long-term stability advantage of atomic clocks.
- Measurement noises of different intensities exert differentiated impacts on the final clock steering performance, and this study has completed the corroborative analysis of the corresponding action mechanisms.

**What are the implications of the main findings?**

- Through targeted simulation validation, the generated clock-steered time signal features superior stability in both the short and long term, which is expected to effectively improve the overall performance of the single-satellite time reference.
- The relevant conclusions can provide clear practical references for selecting appropriate clock steering schemes under different noise conditions, so as to guarantee the stable operation of single-satellite time benchmarks.

**Abstract:**

Clock steering, the core time-frequency technology for high-precision single-satellite time reference generation in global navigation satellite systems, can effectively combine the excellent short-term stability of Oven-Controlled Crystal Oscillators (OCXOs)—whose top performance indicators have already surpassed many space-borne atomic clocks in recent years—with the superior long-term stability of atomic clocks, to obtain time signals with optimal full-range stability. This paper proposes a novel clock steering scheme that integrates an adaptive variational Bayesian Kalman filter and a Proportional-Integral-Derivative (PID) automatic controller: the filter constructs a separable variational approximation for the joint posterior distribution of clock states and measurement noise parameters, to achieve real-time adaptive estimation of noise at each timestamp, while the PID controller performs closed-loop fine adjustment on the output frequency. Comparative simulations with the classic Linear Quadratic Gaussian (LQG) control scheme verify that the proposed method can generate steered time signals with better stability performance in both short-term and long-term dimensions. This work further investigates the influence of measurement noise at different intensity levels on clock steering performance and conducts corresponding mechanism analysis supported by quantitative data. The proposed scheme and conclusions can provide a reliable reference for selecting appropriate clock steering strategies under different noise conditions.

## 1. Introduction

Thanks to the continuous development of high-stability frequency technology, the short-term stability of top-tier crystal oscillators has reached levels of $6\times 10^{-14}$/1 s, $5\times 10^{-14}$/10 s, and $6\times 10^{-14}$/100 s. Notably, the technical route of leveraging the excellent short-term stability of crystal oscillators to complement the outstanding long-term stability of atomic clocks has become the mainstream single-satellite time reference solution in all major global GNSS systems, which realizes high-precision time reference generation on single satellites by adopting mature clock steering algorithms.

However, due to the inherent large long-term drift characteristic of crystal oscillators, cumulative time errors are easily accumulated during long-term operation, resulting in poor mid-term and long-term stability performance. Meanwhile, the short-term stability of existing atomic clocks is generally at the $1\times 10^{-13}$ level, whereas their long-term stability can reach $1\times 10^{-15}$ [1]. Considering the comprehensive advantages of crystal oscillators in terms of size, weight, power consumption, and cost, retaining the superior short-term stability of crystal oscillators and steering their long-term output characteristics to traceable atomic clock references or high-precision GNSS time signals is an effective technical path to further improve the overall performance of space-borne navigation and timing systems [2].

Clock steering technology is the core means of achieving high-precision clock synchronization and is particularly important in distributed systems that require high stability and reliability [3,4,5,6]. The fundamental principle underlying this technology is to correct the local clock through an external high-precision time source (such as a GNSS signal or a better clock). Mostly, in the literature, clock steering is intended to significantly improve the stability of the clock to be steered. The primary process of clock synchronization consists of two steps. Firstly, external signals are decoded and compared with the local clock to obtain an estimate of the clock offset. Secondly, a control algorithm is used to adjust the phase or frequency of the local clock to synchronize it with an external high-precision time source. In the first step, the clock offset measurement data are frequently characterized by substantial jitter, a consequence of factors such as measurement noise and the inherent noise characteristics of the atomic clock. Consequently, methodologies such as Kalman filtering [7,8] are required to process the noise. In the subsequent step, the implementation of automatic control algorithms such as LQG control [9] is imperative. The filter and controller operate in tandem, iterating until convergence to ultimately form an optimal synchronization system.

In recent decades, a considerable body of literature has emerged on the subject of clock steering technologies. In [10], Farina et al. described three common techniques: signal-based open-loop compensation, the GPS bang-bang method, and LQG control. Signal-based open-loop compensation methods are typically characterized by their direct calculation of frequency adjustment values. This calculation is made by measuring the phase difference between the clock and a reference standard (such as UTC) [11]. This method is straightforward to implement and utilizes minimal computational resources. The GPS bang-bang method is initiated by a preset threshold, thereby triggering frequency adjustment. In instances where the phase error exceeds the threshold, a fixed amplitude frequency step correction is applied. In [12], the GPS bang-bang method was designed to adjust the GPS time to UTC (USNO). The LQG method integrates the Kalman filter and linear quadratic regulator to enhance the phase, frequency, and control energy efficiency of the clock in noisy environments [10]. This method is based on balancing the convergence speed and control amplitude through parameter adjustments [9]. The application of pole placement technology to clock steering has also been recently explored [13]. The pole placement (PP) method sets the pole position of the system directly, thereby controlling the response speed and dynamic characteristics of the clock. The primary benefit of this approach is the simplicity of the parameter optimization process, which involves configuring the target pole position. This configuration is straightforward to implement and adjust. In [14], Marion et al. conducted a comparative analysis of the performance of pole placement techniques and LQG control techniques, concluding that there was no significant difference between the two. In 2019, Matsakis unified the two proportional steering strategies for controlled clocks, PP and LQG, to quantify their effects on clock behavior and state estimation [15]. In 2020, Matsakis applied the PID controller for controlling clocks. The PID approach can protect against nonstochastic errors but may result in greater variance in the PFS (phase, frequency and steers) [16].

Concurrently with the advancements in atomic clock synchronization and GNSS systems, a variety of advanced adaptive control and modern estimation algorithms have emerged. For instance, Liu et al. [17] introduced an adaptive Kalman filtering algorithm utilizing the Autocovariance Least Squares (ALS) method for rubidium clock disciplining. In the realm of multi-GNSS, Fu et al. [18] developed a computationally efficient quality control method that significantly enhances real-time clock estimation accuracy by optimizing observation weights. Additionally, Muflihah et al. [19] proposed a two-stage adaptive filtering approach that combines quality scoring with a Kalman filter to improve time synchronization stability.

While these methods have demonstrated superior performance under specific conditions, the primary focus of this study is to validate the advantages of combining two stability metrics through clock steering. Given the foundational status and widespread application of classical LQG control in the field of clock steering, it was selected as the primary baseline for this work. Although a direct comparison with these specific state-of-the-art algorithms was not conducted, the proposed method demonstrates unique value in maintaining high performance while preserving a concise algorithmic structure. The comparison with the classical LQG control sufficiently validates the effectiveness of our strategy in improving timekeeping accuracy.

Although the existing literature has explored various clock steering techniques, the volume of targeted research that combines the short-term stability of oscillators and the long-term stability of atomic clocks remains relatively limited. Relevant studies with similar goals, along with the proposed method, are summarized in Table 1. Due to the difference in environmental conditions and research purposes, it is difficult to make a direct and precise numerical comparison. Moreover, there are hardly any studies on the effects of different levels of measurement noise on clock steering. The purpose of this study is to propose a clock steering method and demonstrate through simulation experiments that it can retain the short-term stability of one clock while benefiting from the long-term stability of another. In Section 4, the proposed method presents better performance than LQG control. Additionally, the impact of different levels of measurement noise on the two steering techniques is studied. The conclusion provides guidance for selecting the proper method under different noise conditions.

While classical control methods for atomic clocks have laid the foundation for GNSS operations, recent research has increasingly focused on adaptive estimation and ultra-rapid synchronization techniques to meet the demands of real-time applications.

For instance, modern time synchronization now relies heavily on ultra-rapid regimes to calculate ephemeris and clock corrections with minimal latency, as demonstrated in recent studies on GLONASS and GPS systems [22]. Furthermore, the accuracy of these estimations has been significantly improved through multi-GNSS precise point positioning (PPP), where the consistency of orbit and clock products across different analysis centers is rigorously assessed [23].

Additionally, understanding the intrinsic stability of satellite clocks remains crucial. Recent analyses utilizing high-rate observation data have provided deeper insights into how reference clock types influence short-term stability, offering a more refined perspective than traditional averaging methods [24]. These modern approaches complement classical control theories by addressing the complexities of current multi-constellation environments.

This paper is organized as follows. In Section 2, we will introduce the relevant work on the proposed steering technique. Then, in Section 3, we will introduce the data sources and experimental methods used in the simulation experiments. In Section 4, we will discuss the experimental results.

## 2. Materials and Methods

In Section 2, we will describe the basics of the proposed steering method, including the clock model, the variational Bayesian Kalman filter for atomic clocks, the PID control technology and the method for experiments.

### 2.1. Clock Model and Variational Bayesian Kalman Filter

The Kalman filter (KF) is employed to estimate the phase, frequency, and frequency drift of a single atomic clock. During the clock steering process, the Kalman filter is a valuable tool for extracting more precise clock difference estimates from observations of clock differences that are subject to noise [25,26,27]. The standard Kalman filter for atomic clocks utilizes a constant measurement noise variance, a value that is empirically determined. This constant value is frequently recalibrated in response to changes in the surrounding environment. The variational Bayesian Kalman filter [28] of the atomic clock optimizes the estimated minimum mean square error matrix by adding a Bayesian adaptive factor, thereby enabling dynamic estimation of measurement noise variance and convergence to the optimal model in the iterative calculation of Algorithm 1.
**Algorithm 1.** Variational Bayesian Kalman filter for atomic clocksClock State Prediction: Predict state:${\hat{x}}^{-}\left(t\right)=\phi \left(\delta \right)\hat{x}(t-\delta )$Predict MMSE matrix:$C^{-}(t)=\phi \left(\delta \right)C(t-\delta )\phi {\left(\delta \right)}^{T}+Q\left(\delta \right)$Adaptive Measurement Noise Estimation: Predict noise parameters:$\alpha^{-}\left(t\right)=\rho \;*\;\left[\alpha \left(t-1\right)+1\right]$ $\beta^{-}\left(t\right)=\rho \;*\;\beta (t-1)$Estimate measurement noise variance:$R\left(t\right)=\beta (t-1)/\alpha (t-1)$Update noise parameters:$\alpha \left(t\right)=\alpha^{-}\left(t\right)$ $\beta \left(t\right)=\beta^{-}\left(t-1\right)+{\left(y\left(t\right)-H\hat{x}\left(t-1\right)\right)}^{2}+HC\left(t\right)H^{T}$Clock State Estimation Update: Update MMSE matrix:$C\left(t\right)=C^{-}\left(t\right)-C^{-}(t)H^{T}{\left(HC^{-}\left(t\right)H^{T}+R\left(t\right)\right)}^{-1}HC^{-}(t)$Update Kalman gain:$K(t)=C^{-}(t)H^{T}{\left(HC^{-}(t)H^{T}+R\left(t\right)\right)}^{-1}$Upstate clock state estimation:$\hat{x}\left(t\right)={\hat{x}}^{-}\left(t\right)+K(t)\left[y\left(t\right)-H{\hat{x}}^{-}\left(t\right)\right]$

In the case of a single clock, the stochastic differential equation (SDE) that describes the state of an atomic clock is as follows [29,30]:

(1) $$x\left(t\right)=\phi \left(\delta \right)x\left(t-\delta \right)+w\left(t,\delta \right)$$

where δ denotes the sampling interval, $w\left(t,\delta \right)\;$ denotes the Gaussian process noise, and the state transition matrix $\phi \left(\delta \right)$ is defined as follows:

(2) $$\phi \left(\delta \right)=\left[\begin{array}{ccc} 1 & \delta & \delta^{2}/2\\ 0 & 1 & \delta \\ 0 & 0 & 1 \end{array}\right]$$

The observation equation for the discrete-time clock state is as follows:

(3) $$y\left(t\right)=Hx\left(t\right)+r\left(t\right)$$

where $H=\left[\begin{array}{ccc} 1 & 0 & 0 \end{array}\right]$ denotes the observation vector and $r\left(t\right)$ denotes the Gaussian measurement noise at time *t*. The equation used to predict the state of the atomic clock at time t is as follows:

(4) $${\hat{x}}^{-}\left(t\right)=\phi \left(\delta \right)\hat{x}(t-\delta )$$

where $\hat{x}\left(t-\delta \right)$ denotes the Kalman filtered estimate of the clock state at time t−δ and ${\hat{x}}^{-}\left(t\right)$ denotes the prediction of the clock state at time t using the state transfer matrix.

The minimum mean square error matrix of the prediction is as follows:

(5) $$C^{-}(t)=\phi \left(\delta \right)C(t-\delta )\phi {\left(\delta \right)}^{T}+Q\left(\delta \right)$$

In the equation above, C(t−δ) denotes the minimum mean square error matrix for clock state estimation at time t−δ, and $Q\left(\delta \right)$ denotes the covariance matrix for the clock noise:

(6) $$Q\left(\delta \right)=\left(\begin{array}{ccc} q_{1}\delta +\frac{1}{3}q_{2}\delta^{3}+\frac{1}{20}q_{3}\delta^{5} & \frac{1}{2}q_{2}\delta^{2}+\frac{1}{8}q_{3}\delta^{4} & \frac{1}{6}q_{3}\delta^{3}\\ \frac{1}{2}q_{2}\delta^{2}+\frac{1}{8}q_{3}\delta^{4} & q_{2}\delta +\frac{1}{3}q_{3}\delta^{3} & \frac{1}{2}q_{3}\delta^{2}\\ \frac{1}{6}q_{3}\delta^{3} & \frac{1}{2}q_{3}\delta^{2} & q_{3}\delta \end{array}\right)$$

where $q_{1}$, $q_{2}$ and $q_{3}$ represent the diffusion coefficients of the atomic clock’s power-law noise, corresponding to white frequency modulation (WFM), random-walk frequency modulation (RWFM), and random-run frequency modulation (RRFM), respectively. The enhancement of the variational Bayesian adaptive Kalman filter over the original method entails augmenting the iterative tuning of the measurement noise variance.

Furthermore, the optimization of the minimum mean square error matrix of the estimate is imperative to ensure superior estimation accuracy. A theoretical explanation can be seen in [28], which proposes a heuristic dynamic for the noise variances. The measurement noise variance parameters $\alpha^{-}\left(t\right)$ and $\beta^{-}\left(t\right)$ are predicted as follows:

(7) $$\alpha^{-}\left(t\right)=\rho \;*\;\left[\alpha \left(t-1\right)+1\right]$$

(8) $$\beta^{-}\left(t\right)=\rho \;*\;\beta (t-1)$$

where ρ denotes the propagation coefficient of the noise variance parameter. The estimate of measurement noise variance is given by the updated $\alpha \left(t-1\right)$ and $\left(t-1\right)$:

(9) $$R\left(t\right)=\beta (t-1)/\alpha (t-1)$$

With each filtering update to $\alpha \left(t-1\right)$ and $\beta \left(t-1\right)$, the adaptive noise variance R(t) converges to an optimal value that is close to the true value, ultimately yielding the optimal filter.

The minimum mean square error (MMSE) matrix of the estimate is updated as:

(10) $$C\left(t\right)=C^{-}\left(t\right)-C^{-}(t)H^{T}{\left(HC^{-}\left(t\right)H^{T}+R\left(t\right)\right)}^{-1}HC^{-}(t)$$

The measurement noise variance parameters $\alpha \left(t\right)$ and $\beta \left(t\right)$ are updated as:

(11) $$\alpha \left(t\right)=\alpha^{-}\left(t\right)$$

(12) $$\beta \left(t\right)=\beta^{-}\left(t-1\right)+{\left(y\left(t\right)-H\hat{x}\left(t-1\right)\right)}^{2}+HC\left(t\right)H^{T}$$

The Kalman gain is updated as:

(13) $$K(t)=C^{-}(t)H^{T}{\left(HC^{-}(t)H^{T}+R\left(t\right)\right)}^{-1}$$

where $R\left(t\right)$ denotes the covariance of the measurement noise. After correction, the Kalman estimation equation for the atomic clock state at time *t* is finally given as follows:

(14) $$\hat{x}\left(t\right)={\hat{x}}^{-}\left(t\right)+K(t)\left[y\left(t\right)-H{\hat{x}}^{-}\left(t\right)\right]$$

where $y\left(t\right)$ represents the measurement of the clock at time t.

### 2.2. PID Controller for Steering Clocks

The PID controller is linear [31]. Its primary advantage lies in its ability to achieve a fast response to system errors, eliminate steady-state errors, and suppress system oscillations through three adjustments: proportional, integral, and differential. These three adjustments take into account the response speed, accuracy, and stability of the system [32,33,34]. The PID controller calculates the control deviation $e\left(t\right)$ based on the setpoint $r\left(t\right)$ and the actual output value $f\left(t\right)$:

(15) $$e\left(t\right)=r\left(t\right)-f\left(t\right)$$

In this article, given the value $r\left(t\right)=0$, the actual output value $f\left(t\right)$ refers to the Kalman estimate of frequency, which is the second element of $\hat{x}\left(t\right)$. The PID control output $u\left(t\right)$ is obtained by performing proportional, integral, and derivative operations on the control deviation $e\left(t\right)$ in that order. $e\left(t\right)$ is rewritten as:

(16) $$e\left(t\right)=-{\hat{x}}_{2}\left(t\right)$$

where $-{\hat{x}}_{2}\left(t\right)$ denotes the value of the frequency estimate.

To reduce the impact of the phase noise, the phase estimates will not be used at all, which means the proposed method will adopt only frequency adjustments, no phase adjustments. The proportional term $P\left(t\right)$ in the control output is as follows:

(17) $$P\left(t\right)=e\left(t\right)\;*\;K_{p}$$

where $K_{p}$ is the parameter used for calculating the proportional term and $K_{i}$ and $K_{d}$ are used for calculating the integral and derivative gains, respectively. The integral term $I\left(t\right)$, derivative term $D\left(t\right)$, and the PID control output $u\left(t\right)$ are as follows:

(18) $$I\left(t\right)=I\left(t\right)+e\left(t\right)\;*\;K_{i}$$

(19) $$D\left(t\right)=K_{d}\;*\;\left(e\left(t\right)-e\left(t-1\right)\right)$$

(20) $$u\left(t\right)=P\left(t\right)+I\left(t\right)+D\left(t\right)$$

**Figure 1:**
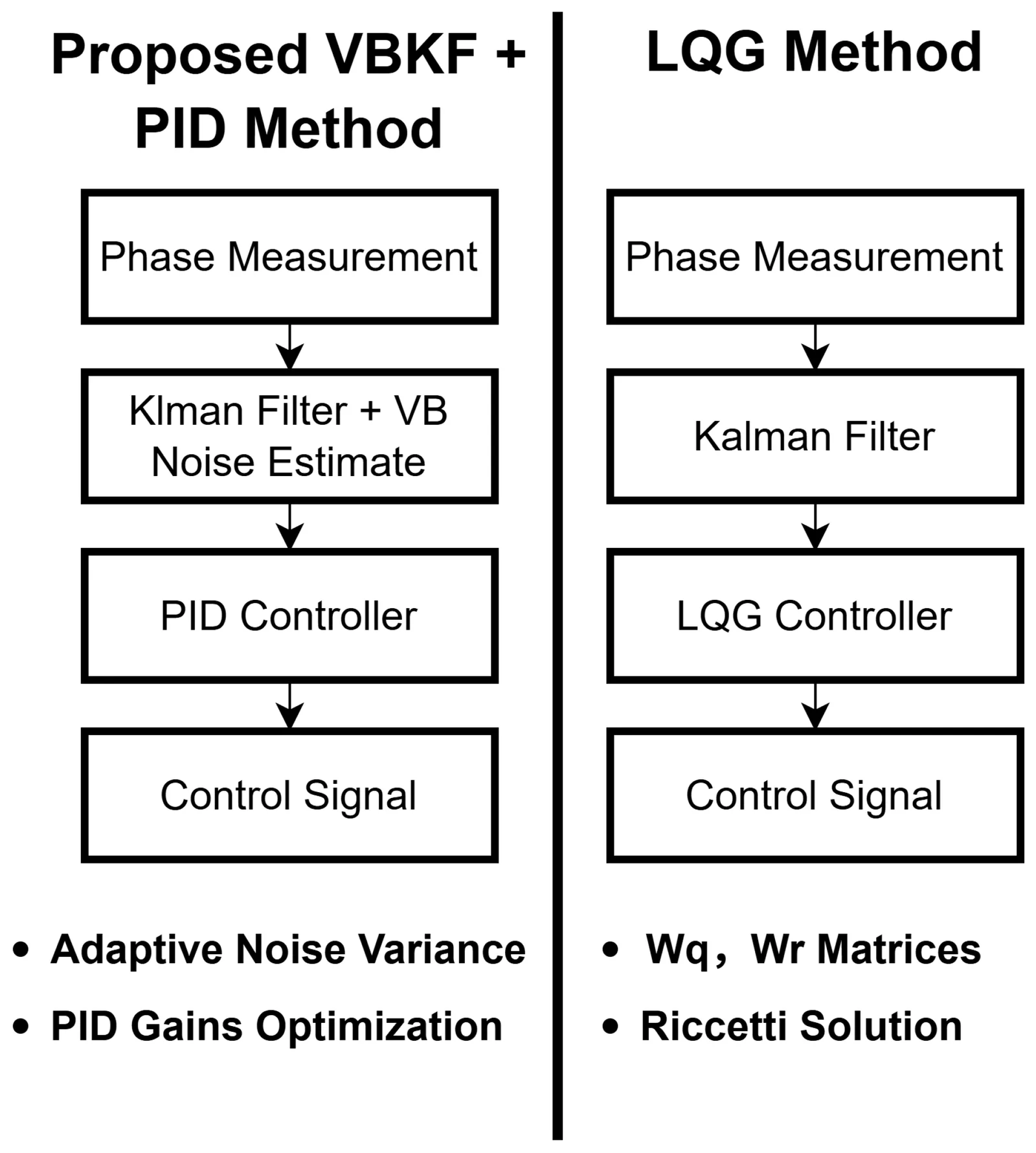
A general schematic diagram of two methods.

In this study, the PID parameters for phase and frequency were held constant; although they can be tuned to adjust the response velocity and fluidity, Figure 1 further illustrates the structural distinction between the proposed VBKF+PID and LQG methods, highlighting the former’s use of adaptive noise covariance versus the latter’s reliance on fixed weighting matrices.

### 2.3. Methods for Experiments

#### 2.3.1. Data Generation

**Figure 2:**
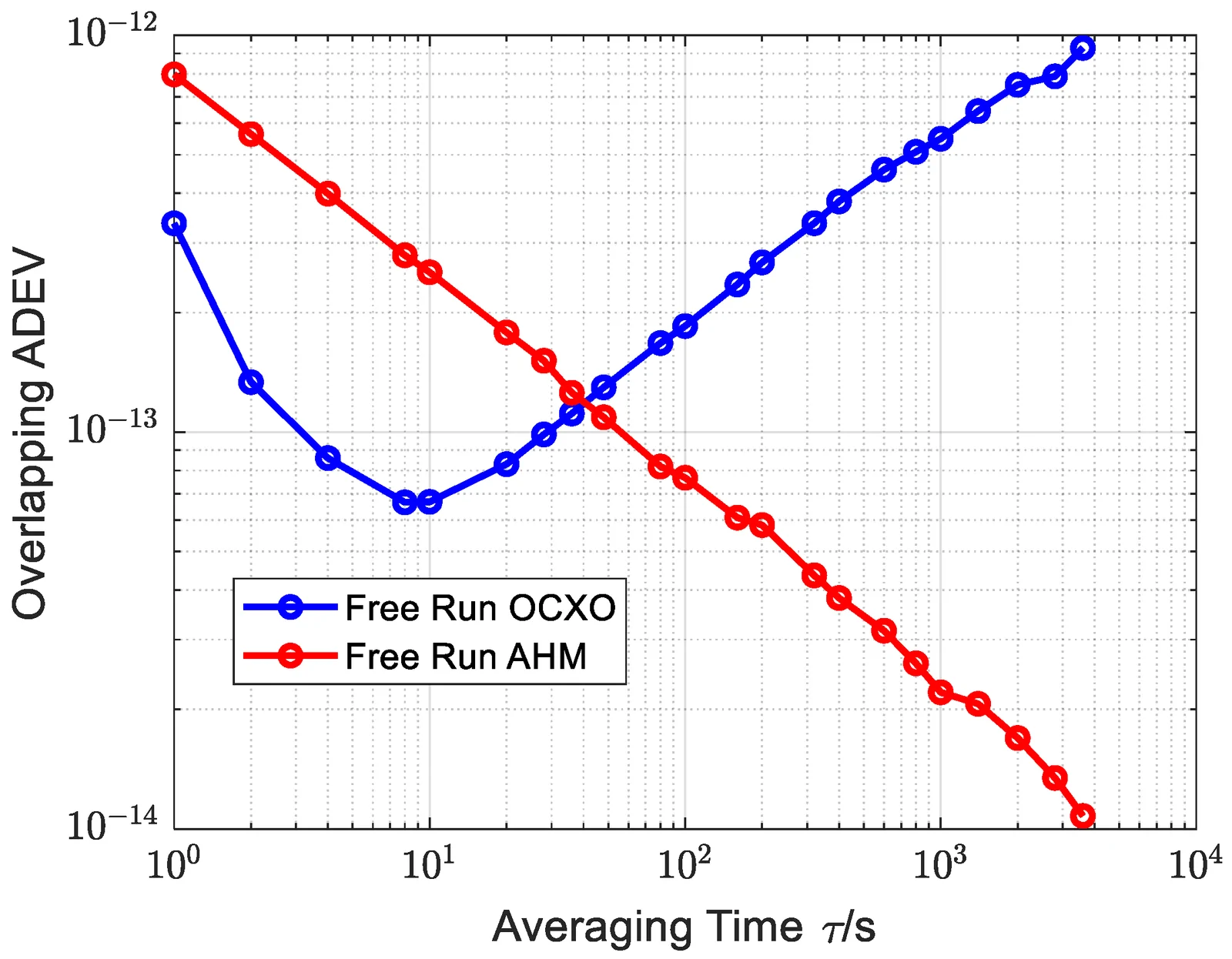
Stability evaluation of free−run OCXO and AHM.

The proposed steering method and LQG method were run with simulated atomic clock data. The clock model employed for the simulation has been derived from [7]. The diffusion coefficient values utilized are enumerated in Table 2, which referenced [35,36,37]. The simulation duration is set to 16 h with a measurement noise of 0.5 ps. Figure 2 illustrates the stability of the free-running OCXO and active hydrogen maser (AHM).

#### 2.3.2. Parameter Selection

The parameters of the LQG controller and PID controller are delineated in Table 3 and Table 4, where the parameters of the LQG controller are derived from [9,14]. The parameters of the PID controller are obtained by minimizing the cost function. In this paper, the PID controller is combined with the variational Kalman filter to produce satisfactory results.

To obtain the optimal LQG control frequency steering value $u_{LQG}\left(t\right)$, it is necessary to ensure that the cost function J is minimized [9]:

(21) $$J=E\left[\sum_{t}\left[x^{T}\left(t\right)\right]W_{Q}x\left(t\right)+u_{LQG}^{T}\left(t\right)W_{R}u_{LQG}\left(t\right)\right]$$

where $W_{Q}$ and $W_{R}$ are the parameters of the LQG. The LQG control frequency steering value is calculated as:

(22) $$u_{LQG}\left(t\right)=-{\hat{G}}_{0}x\left(t\right)$$

The LQG gain ${\hat{G}}_{0}$ is obtained by:

(23) $${\hat{G}}_{0}={\left(B^{T}{\hat{K}}_{0}B+W_{R}\right)}^{-1}B^{T}{\hat{K}}_{0}\Phi$$

where $B=\left[\begin{array}{c} \delta \\ 1 \end{array}\right]$, $\Phi =\left[\begin{array}{cc} 1 & \delta \\ 0 & 1 \end{array}\right]$, and ${\hat{K}}_{0}$ is obtained by solving the discrete-time steady-state Riccati equation:

(24) $${\hat{K}}_{0}=\Phi^{T}{\hat{K}}_{0}\Phi +W_{Q}-\Phi^{T}{\hat{K}}_{0}B{\left(B^{T}{\hat{K}}_{0}B+W_{R}\right)}^{-1}B^{T}{\hat{K}}_{0}\Phi$$

$W_{Q}$ and $W_{R}$ can be selected using the following equation [33]. When the function value is zero, the optimal values of the parameters are obtained:

(25) $$f={\left(\delta g_{x}+g_{y}-2\right)}^{2}-4\left(1-g_{y}\right)$$

where $g_{x}$ and $g_{y}$ are the phase gain and frequency gain in ${\hat{G}}_{0}$, respectively. The corresponding function values for the four sets of LQG parameters are $-1.97\times 10^{-4}$, $-6.32\times 10^{-8}$, $9.69\times 10^{-4}$ and −0.0173. All of them are in close proximity to zero. Despite the discrepancy in the experimental setup between this study and [9] (with sampling intervals of 1 s and 3600 s, respectively), all three trials of parameters were still employed for the simulation. Additionally, we added a set of LQG parameters from [14] (Trial 4) because the two experiments exhibit greater similarity (both with a sampling interval of 1 s).

As for the PID controller, optimal parameters can also be obtained by minimizing a cost function, which can be expressed as:

(26) $$J_{1}=f\left({\bar{K}}_{p},{\bar{K}}_{i},{\bar{K}}_{d}\right)$$

Since our objective is to utilize the short-term stability and long-term stability of the two clocks, $J_{1}$ is defined as:

(27) $$J_{1}=\sum_{i}\frac{\sigma \left(i\right)}{\sigma_{OCXO}\left(i\right)}+\sum_{j}\frac{\sigma \left(j\right)}{\sigma_{AHM}\left(j\right)}$$

where i=1,4,10; j=100,400,1000; and $\sigma \left(t\right),\;\sigma_{OCXO}\left(t\right)$ and $\sigma_{AHM}\left(t\right)$ denote the overlapping ADEV of the steered OCXO, the free-running OCXO and the free-running AHM at time t. Through searching, the values of $K_{p},K_{i}$ and $K_{d}$ are obtained:

(28) $$\left[\begin{array}{ccc} K_{p} & K_{i} & K_{d} \end{array}\right]=arg_{min}f\left({\bar{K}}_{p},{\bar{K}}_{i},{\bar{K}}_{d}\right)$$

Additionally, considering the impact of the control intervals on the steering results, we conducted searches using different control intervals, with the outcomes presented in Table 4. The results indicate that altering the control intervals affects the optimal parameters.

**Figure 3:**
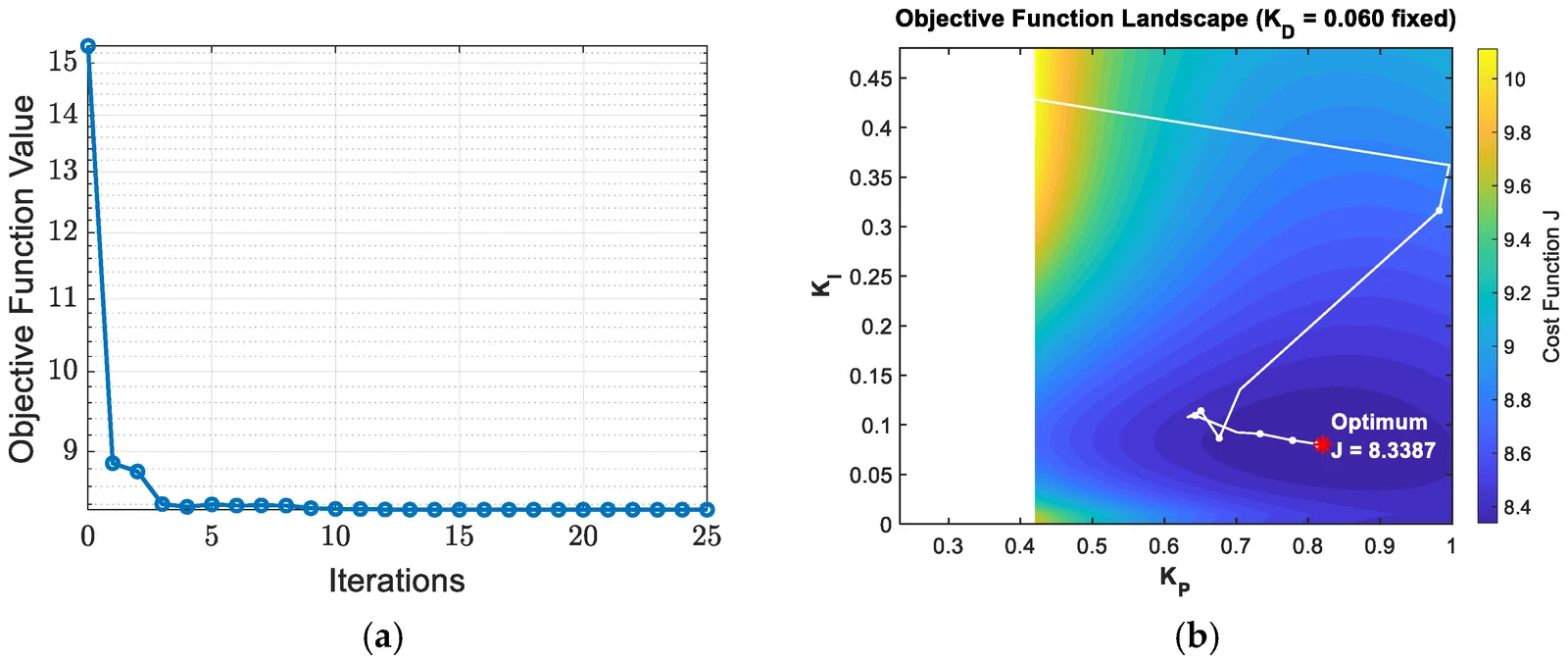
Simulation results for the parameter optimization. (**a**) Convergence curve of the objective function value during the PID parameter optimization process. (**b**) Two-dimensional contour plot of the objective function landscape with respect to $K_{p}$ and $K_{i}$ ($K_{d}$ fixed at its optimal value).

The parameter optimization process is illustrated using the experimental group with a control interval of 20 s as a representative case. The PID parameters were optimized using the fmincon solver in MATLAB R2021B, with the maximum number of iterations set to 500 and the optimality tolerance set to $1\times 10^{-6}$. During the optimization process, the search space for $K_{p}$, $K_{i}$, and $K_{d}$ is constrained within the range of [0, 1]. As shown in Figure 3a, the convergence curve indicates that the algorithm reached the convergence threshold at the 15th iteration, successfully identifying a feasible local optimum that satisfies all constraints. To further elucidate the objective function landscape and parameter coupling, a two-dimensional contour plot of the cost function with respect to $\;K_{p}$ and $K_{i}$ is provided in Figure 3b, with $K_{d}$ fixed at its optimal value. This contour map visually confirms that the identified solution resides within a well-defined minimum basin. The entire optimization procedure was conducted offline; the computational cost is acceptable on standard hardware and does not impede the deployment of the practical control system.

Irrespective of the designated control interval, a Kalman filter is executed once per second to estimate the discrepancy between the OCXO and AHM based on the measured values. For each set of data, two types of curves were plotted: the first type is the frequency stability of the free-run OCXO and AHM; the second type is the frequency stability of the steered OCXO and AHM. The first type of curve demonstrates the performance of OCXO and AHM as standalone entities. The second type of curve illustrates the performance of the steering method. Moreover, the phase difference between the steered OCXO and the free-running AHM is also shown. Once the OCXO and AHM have been synchronized, the phase difference between them will undergo negligible changes, with only minor fluctuations occurring within a small range. This phenomenon is predominantly influenced by white frequency modulation noise. Consequently, when the stability and phase after steering demonstrate characteristics analogous to white noise, it signifies the efficacy of the steering.

#### 2.3.3. Fused Clock Stability Evaluation

While the stability curves (ADEV) provide a standard visualization for frequency stability, they are insufficient for quantitatively evaluating the specific trade-offs in clock combination algorithms. Specifically, ADEV curves describe stability trends but do not offer a scalar metric to assess how effectively a method balances the short-term stability of one source against the long-term stability of another.

To address this limitation, we introduce the index P. Unlike the classical ADEV, the P index is designed to quantify the overall performance of the steering algorithm by capturing the compromise between short-term and long-term stability.

It takes two steps to calculate P:

First, calculate the normalized deviation of overlapping ADEV corresponding to the average time:

(29) $$D\left(i\right)=\left\{\begin{array}{c} \frac{\sigma \left(i\right)-\sigma_{OCXO}\left(i\right)}{\sigma_{OCXO}\left(i\right)},\;\;\;\;\;\;\;\;\;\;\;\;\;1\leq i\leq n\\ \frac{\sigma \left(i\right)-\sigma_{AHM}\left(i\right)}{\sigma_{AHM}\left(i\right)},\;\;\;\;\;n+1\leq i\leq N \end{array}\right.$$

where 1≤i≤n refers to the average time corresponding to the region to the left of the intersection point of the stability curves of the OCXO and the AHM (τ<40) and n+1≤i≤N refers to the average time corresponding to the region to the right of the intersection point of the stability curves of the OCXO and the AHM (τ>40).

Second, to obtain P, calculate the standard deviation of D. The smaller it is, the better the steered signal synthesizes the stability advantages of both methods, aligning more closely with the objective of this study.

With the calculated *p*-value, the fusion degree between the two stability specifications can be quantitatively characterized.

## 3. Results

The results are presented in four parts: first, the performance comparison between VBKF and KF with the controller excluded; second, the simulation results of steering that leverage the stability characteristics of two clocks; third, comparison in time domain; and fourth, the steering results under different noise conditions. Meanwhile, the corresponding LQG control results are also provided. The comparative data show that the proposed method performs better than LQG control in all test scenarios. The variation trends related to noise and the control interval are also presented in the results.

### 3.1. Comparison of VBKF and KF

In Section 3.1, the influence of the controller is eliminated to present the direct performance comparison results between the proposed VBKF method and the conventional KF method [8]. The standard deviation of the residual between the estimated phase value and the reference true phase value is adopted as the evaluation metric to quantify the measurement uncertainty, which corresponds to the core optimization objective of the proposed atomic clock-oriented VBKF for enhancing the filter’s adaptability to time-varying noise environments. Under different noise conditions, the full performance datasets of the two filters are provided. For the conventional KF, two groups of experimental results are reported: one group assigns noise parameters with their real reference values for each independent test, and the other group uniformly sets the noise parameter to a fixed 1 ps across all tests. Furthermore, the complete observation results of the adaptive noise parameter convergence behavior of the proposed VBKF in all test scenarios are also presented.

**Figure 4:**
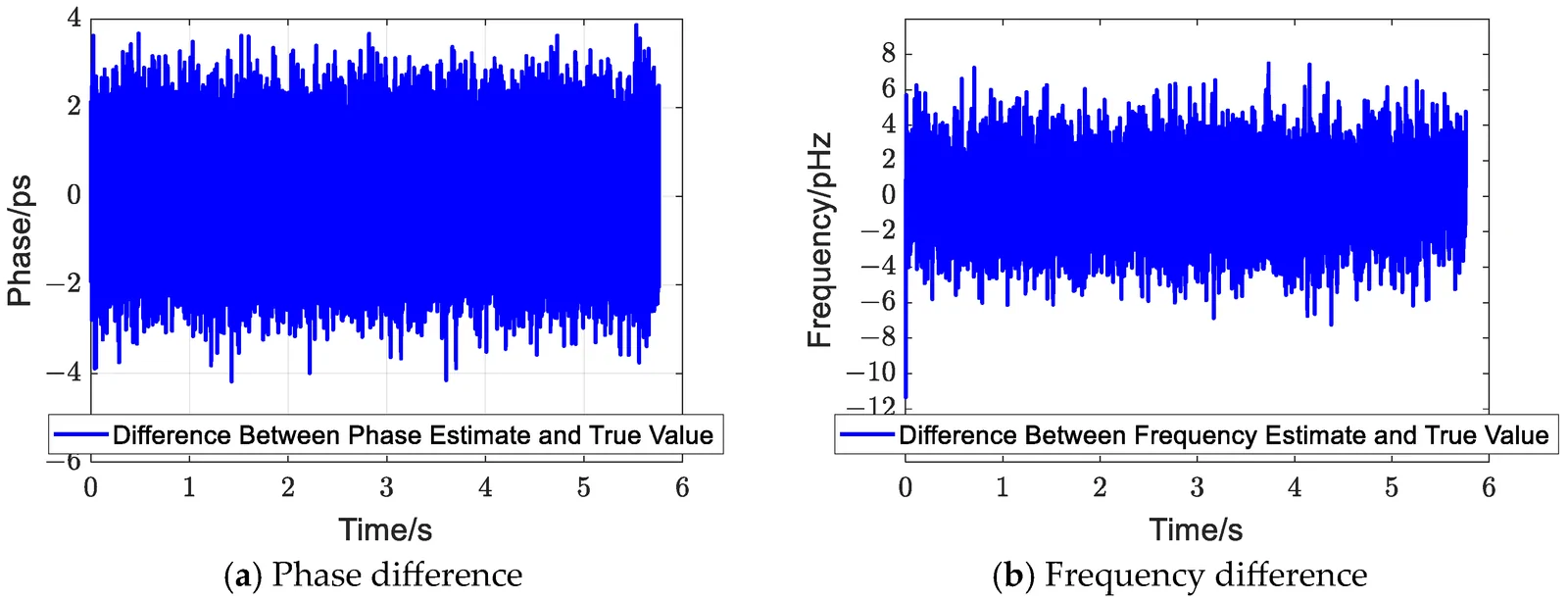
Simulation results for the established KF method with measurement noises of 1 ps (noise parameters correctly set), with phase on the left and frequency on the right.

Figure 4 only presents the simulation results under the measurement noise level of 1 ps with correctly set KF noise parameters, where the left panel demonstrates the phase variation, and the right panel corresponds to the frequency variation. Full sets of supplementary experiments with measurement noise levels of 5 ps and 10 ps under the same KF noise parameter configuration are also completed in this study. The quantitative calculation results indicate that, for test groups with 1 ps, 5 ps and 10 ps measurement noise, the standard deviations of the difference between the estimated phase value and the true phase value are 0.99 ps, 4.01 ps and 5.68 ps, respectively.

**Figure 5:**
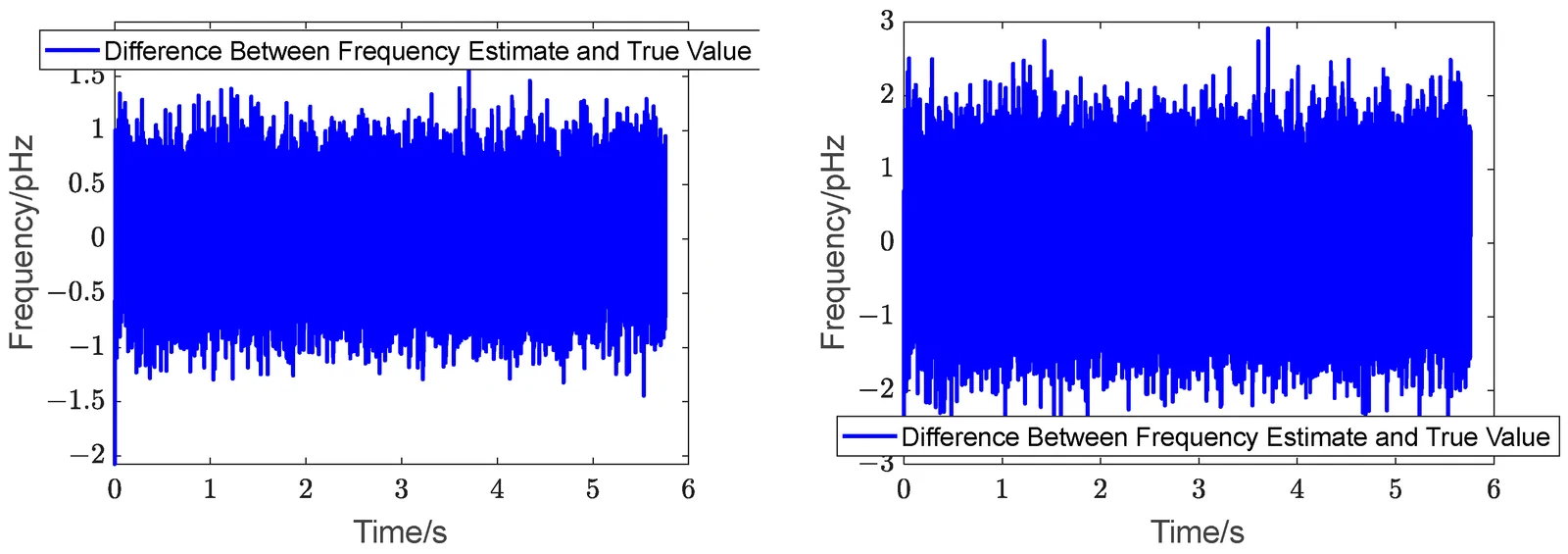
Simulation results for the established KF method with measurement noises of 5 ps and 10 ps (noise parameters set as 1 ps).

As illustrated in Figure 5, the simulation outcomes are presented with the KF noise parameters fixed at 1 ps, while the measurement noise is set at 5 ps and 10 ps, resulting in standard deviations of 4.95 ps and 9.90 ps, respectively. In circumstances where the KF noise parameter and noise magnitude exhibit substantial disparities, the filter’s capacity for noise reduction experiences a notable decline, concomitantly with a substantial augmentation in the estimated uncertainty. This phenomenon is also manifested in the frequency.

**Figure 6:**
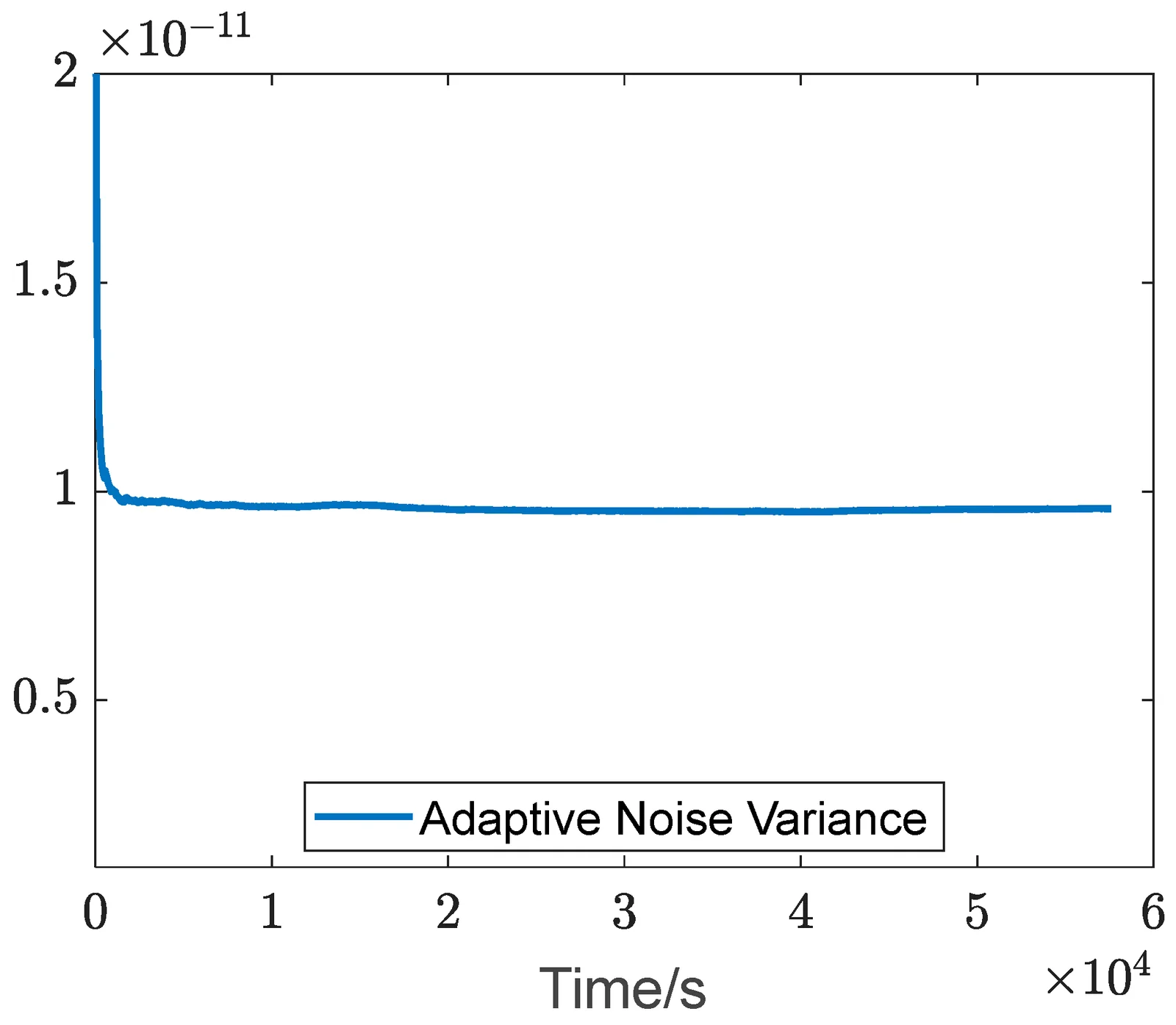
Adaptive noise variance of the VBKF method with measurement noises of 10 ps.

As shown in Figure 6, only the adaptive noise variance result under the 10 ps measurement noise scenario is visualized. The full set of experiments covering 1 ps, 5 ps and 10 ps noise levels have been completed, and the core simulation outputs of the proposed VBKF across all three scenarios are supplemented below: the frequency estimation results for all test cases are not included in this figure, and only the 10 ps case of adaptive noise variance is displayed. The corresponding standard deviations of phase estimation for the three groups are 0.97 ps, 3.76 ps and 5.83 ps, respectively.

Experimental validation demonstrates that the adaptive noise variance in all three test groups converges to values extremely close to the ground-truth noise levels. For the standard Kalman filter (KF), a significant deviation between the pre-set noise parameters and actual noise values leads to a prominent increase in phase estimation uncertainty: when the KF is initialized with a 1 ps preset noise while the true noise level is 10 ps, the phase estimation uncertainty rises drastically, which would introduce severe performance degradation for steering systems relying heavily on frequency estimation outputs. By contrast, the proposed VBKF adaptively adjusts the noise variance and converges it to the accurate value, achieving estimation uncertainty comparable to the standard KF with fully calibrated noise parameters.

### 3.2. To Utilize the Stability Characteristics of Two Clocks

In Section 3.2, the full set of experimental results of the designed steering strategy that leverages the short-term frequency stability of the OCXO and the long-term frequency stability of the AHM are explicitly presented, where the system measurement noise is fixed at 0.5 picoseconds, the 4-s adjustment interval result is independently visualized in a separate figure, and the results corresponding to 10 s, 20 s and 40 s adjustment intervals are collectively plotted in another unified figure. Four independent trials with different LQG control parameter settings are completed, and the inherent performance metrics of free-running OCXO and free-running AHM are supplemented in the corresponding figures as baseline references.

**Figure 7:**
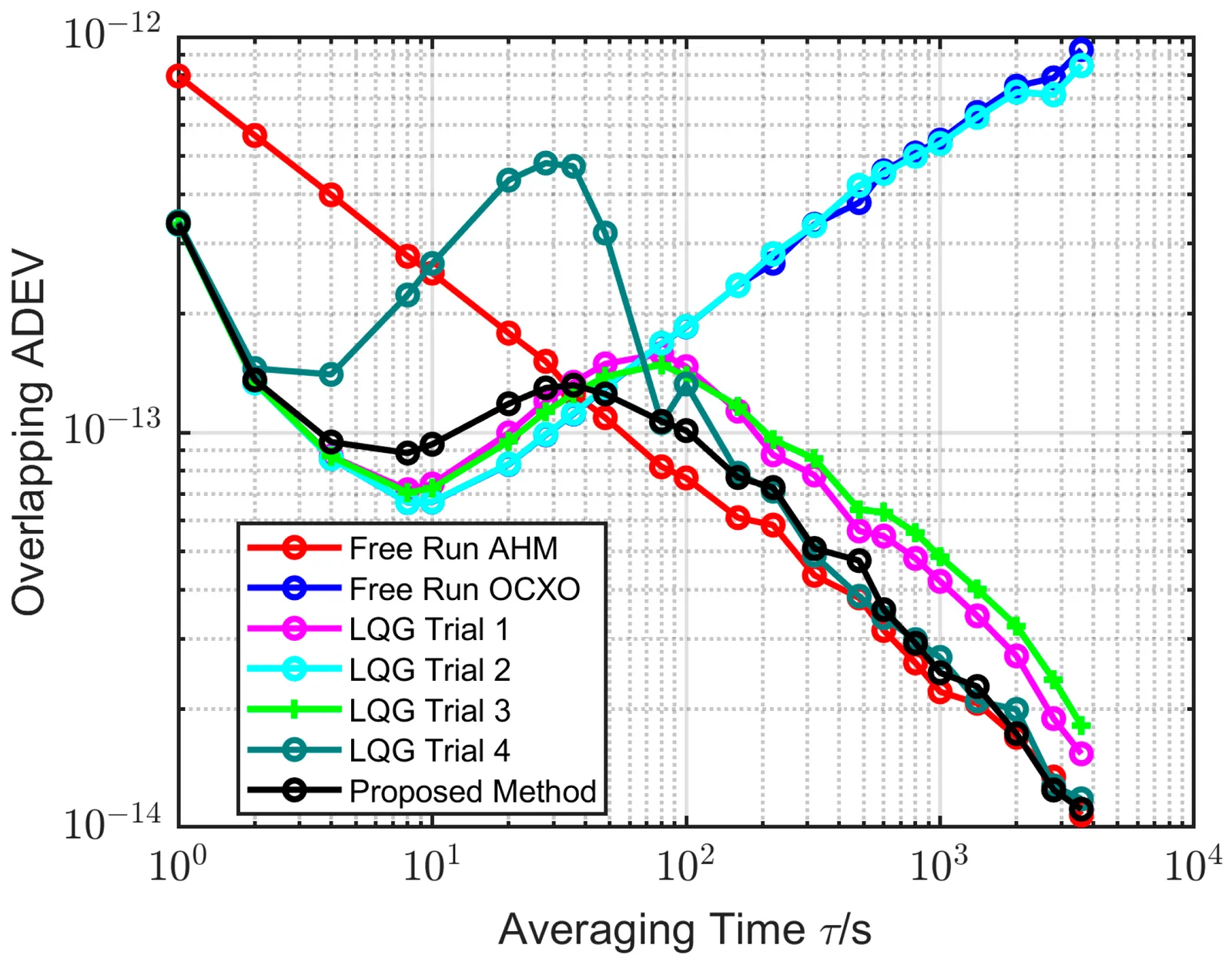
Overlapping ADEV of results of two methods with a control interval of 4 s.

**Figure 8:**
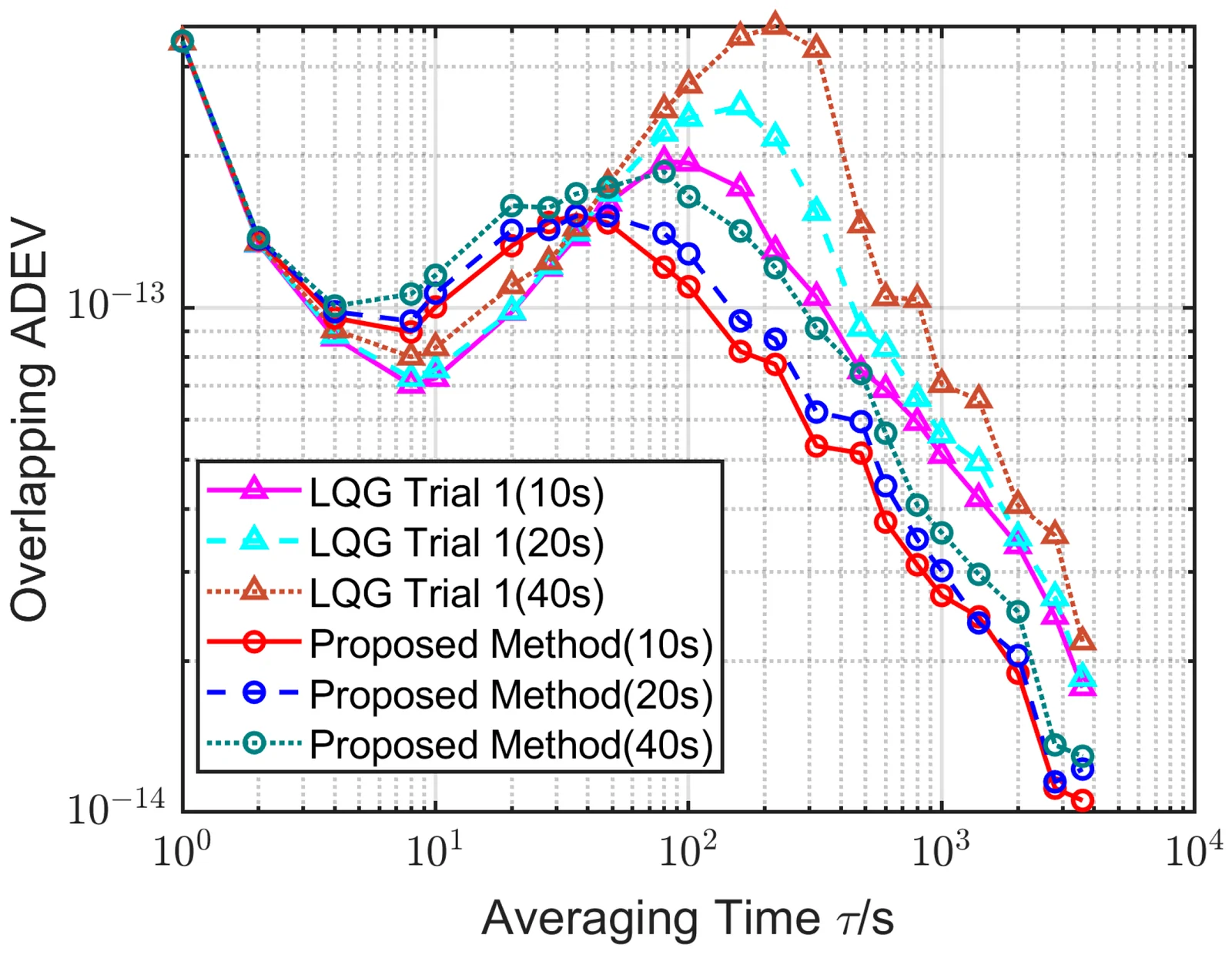
Overlapping ADEV of results of two methods with different control intervals.

Figure 7 and Figure 8 illustrate the performance of both methods under various control intervals. The proposed method consistently achieves optimal performance, yielding *p* = 0.14 at the 4-s interval. In contrast, the LQG controller exhibits high sensitivity to parameter tuning. Among the four LQG trials, Trials 1 and 3 demonstrate the best short-term stability (*p* = 0.43 at the 4-s interval), while Trial 4 achieves the best long-term stability (*p* = 0.44). Notably, Trial 2 fails to provide valid frequency steering due to drastic variations in the calculated LQG gain (G_0_) across different sampling intervals. Furthermore, across all tested intervals, the steered OCXO exhibits regular, opposite fluctuations in its short-term and long-term stability metrics.

Table 5 presents the *p*-values quantifying the similarities in stability between the steered signal and the target (i.e., the short-term stability of the free-running OCXO and the long-term stability of the free-running AHM). Consistent with the preceding experimental results, the data confirm that while LQG control can achieve excellent performance in either short- or long-term stability individually, it fails to balance both metrics simultaneously. This inherent limitation explains the observed *p*-value discrepancy and underscores the substantial advantage of the proposed method, particularly at larger control intervals.

### 3.3. Statistical Analysis

In this subsection, we present a comprehensive statistical analysis of the experimental results, evaluating the mean square error (MSE), standard deviation (STD), 95% confidence intervals (95% CI), and statistical significance.

Table 6 presents the phase MSE values of the simulation results for both methods corresponding to Figure 7 and Figure 8, which measures the synchronization accuracy in the time domain. The phase MSE is denoted as:

(30) $${MSE}_{phase}=\frac{1}{n}\sum_{i=1}^{n}{\left[p_{steered\;clock}\left(i\right)-p_{AHM}\left(i\right)\right]}^{2}$$

where $p_{steeredclock}$ denotes the phase of the steered OCXO and $p_{AHM}$ denotes the phase of the free-running AHM. To obtain the optimal parameters of the proposed method for time synchronization, the cost function $J_{1}$ is changed to $J_{2}$, simply using the phase MSE. The results are presented in Table 6.

Table 7 presents the results of a simulation experiment conducted using real temperature control data, summarizing the statistical performance of the proposed method (PM) against the LQG baseline. Consistent with the experimental setup in Section 3.4, the evaluation validates the robustness of the proposed approach, which maintains a lower standard deviation (13.39 ps vs. 15.89 ps). The observed improvement is statistically significant (*p* < 0.001), indicating that the proposed method offers better consistency compared to the baseline.

### 3.4. Steering Under Different Noises

This section details the simulation results of the steering performance under varying measurement noise levels. We would like to clarify that we did not intend to use simple white noise to represent the complex space noise environment; rather, the objective was to investigate the impact of noise on the algorithm performance under different measurement noise levels and to provide a guideline for parameter selection strategies. To systematically characterize these noise-induced influences, the measurement noise—previously fixed at 0.5 ps—was adjusted to 1 ps, 5 ps, and 10 ps, while all other datasets and parameters remained identical to those specified in Section 4.2. For the LQG control scheme, the parameter sets corresponding to Trial 1 and Trial 4 (which demonstrated superior performance in prior experiments) were selected for validation, with the control interval uniformly set to 4 s to ensure efficiency.

**Figure 9:**
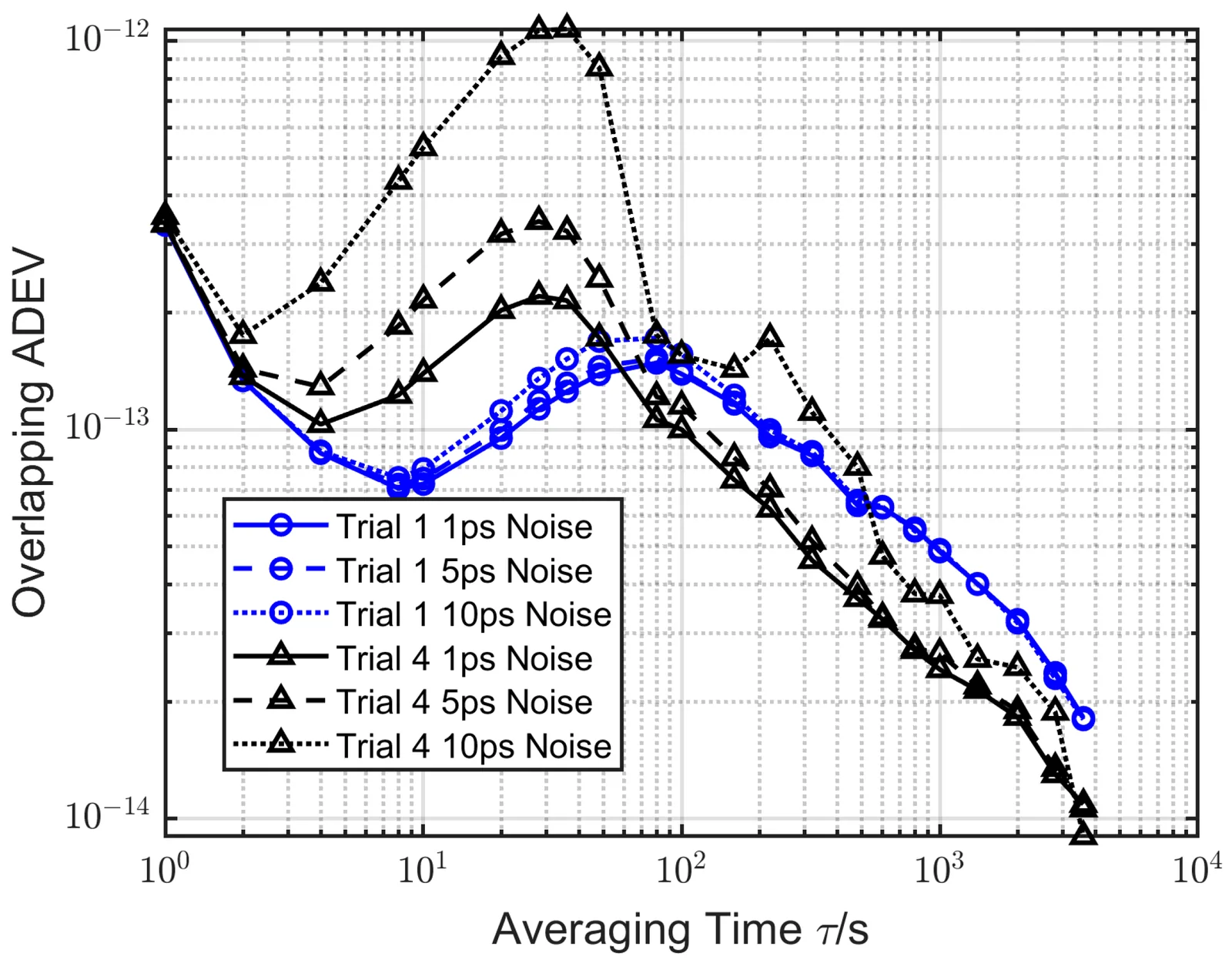
Overlapping ADEV of the results of LQG Trials 1 and 4 under different measurement noise.

**Figure 10:**
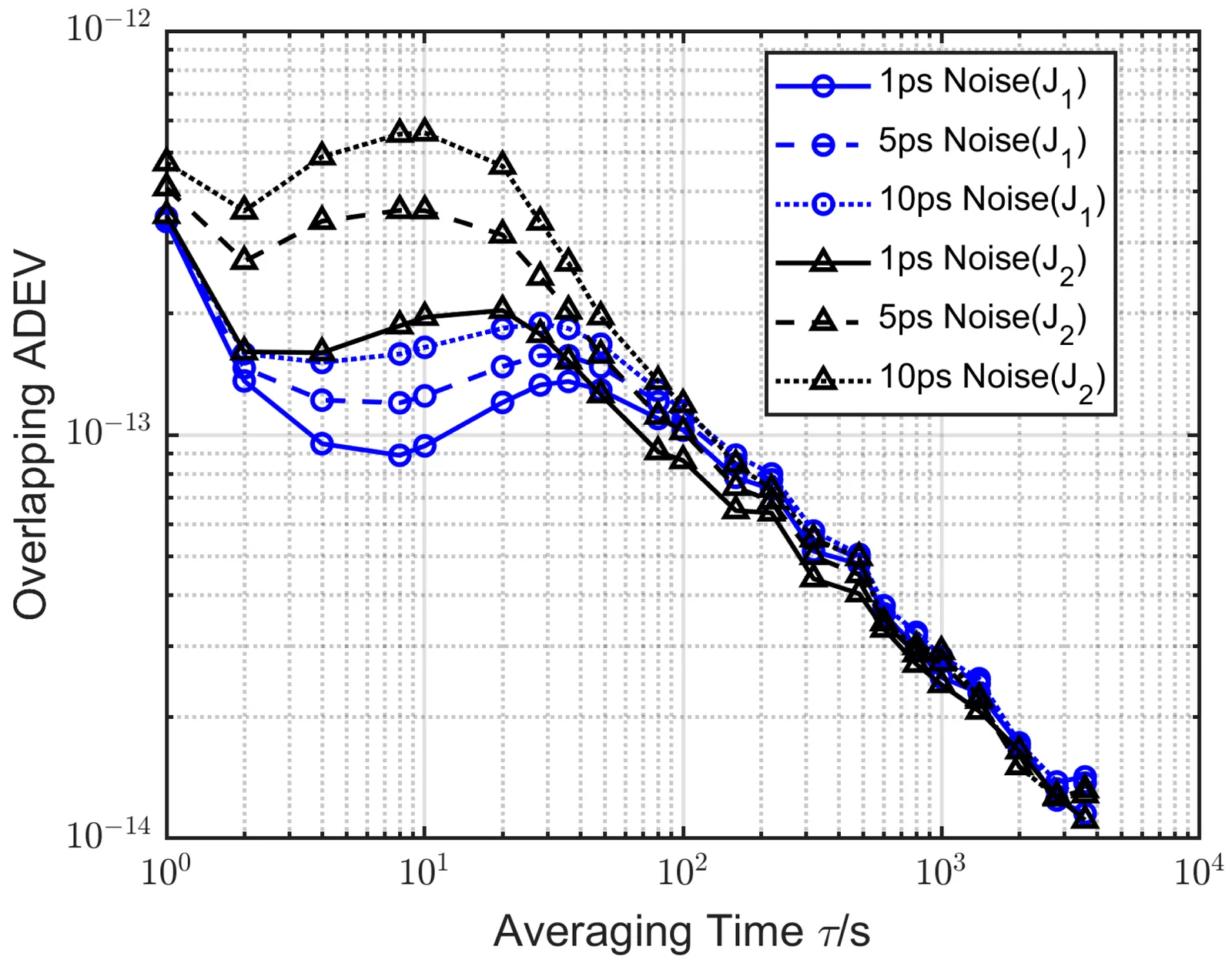
Overlapping ADEV of the results of the proposed method using different cost functions under different measurement noise.

Figure 9 and Figure 10 present the quantitative results under elevated measurement noise. For LQG control, Trial 4 achieves favorable time-domain performance, whereas Trial 1 yields unsatisfactory outputs; notably, the parameter set with higher time-synchronization accuracy exhibits degraded short- and mid-term stability under increased noise excitation, without significant long-term fluctuations. The proposed method demonstrates a consistent response pattern: when optimized via cost function $J_{2}$ for lower phase MSE, its short-term stability degrades more markedly under high-noise conditions, mirroring the variation trend observed in LQG Trial 1.

**Figure 11:**
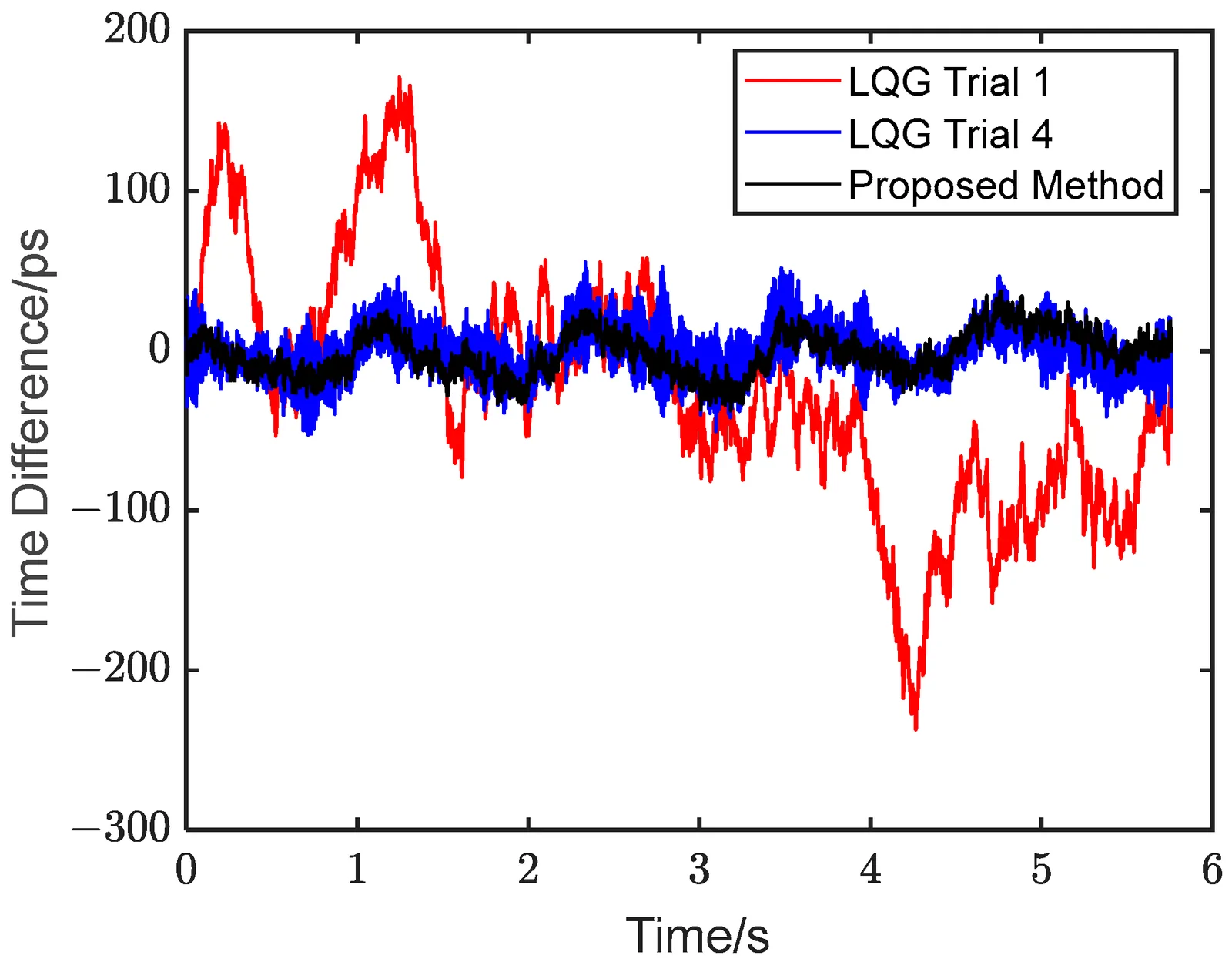
Time synchronization error of steered signal and free-running AHM.

To rigorously evaluate the robustness of the proposed method in practical satellite applications, real-world ground-based temperature control test data obtained from a satellite prototype were utilized for simulation. This dataset faithfully reproduces the thermal variation characteristics of a Low Earth Orbit (LEO) satellite environment. Figure 11 illustrates the comparison of the time synchronization error between the proposed method and the LQG method during the clock steering process. The results demonstrate that the proposed method achieves superior performance (Std = 13.39 ps) compared to the LQG method (Std = 15.89 ps) in the presence of temperature variation noise.

### 3.5. Experimental Results

**Figure 12:**
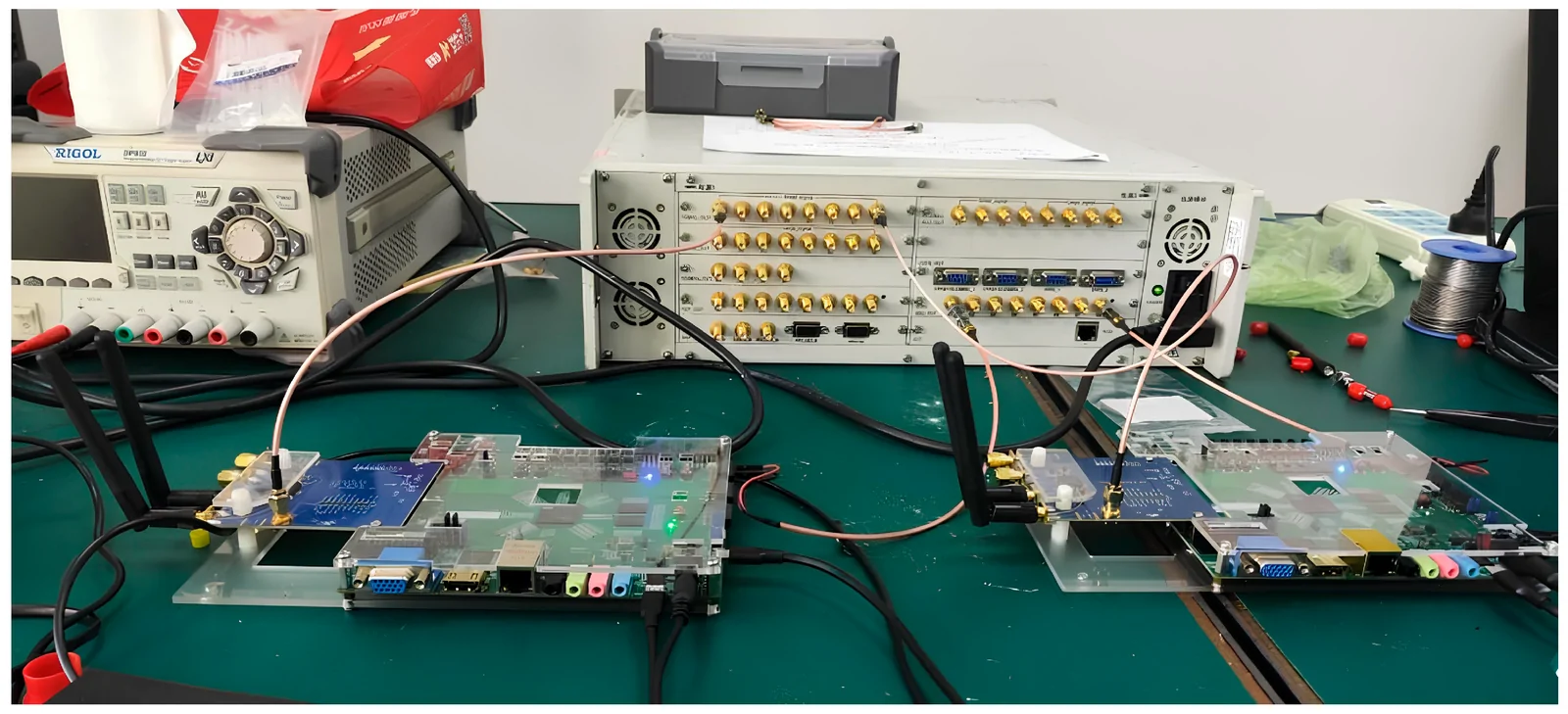
Experimental environment.

This section details the hardware experimental validation. The experimental setup employs an VCH-1003M AHM (Vremya-CH, Nizhny Novgorod, Russia) and an OCXO (Tianao, Chengdu, China) as clock sources. Wireless clock offset measurements are conducted using a platform based on a ZedBoard development board (Avnet Electronics Marketing, Phoenix, AZ, USA), while a phase and frequency micro-jump meter is utilized for frequency steering. Due to the experimental constraints, this study primarily focuses on verifying the proposed method in terms of time synchronization accuracy. The experimental environment is illustrated in Figure 12; however, please note that the visual presentation of certain equipment is omitted in accordance with institutional security regulations.

**Figure 13:**
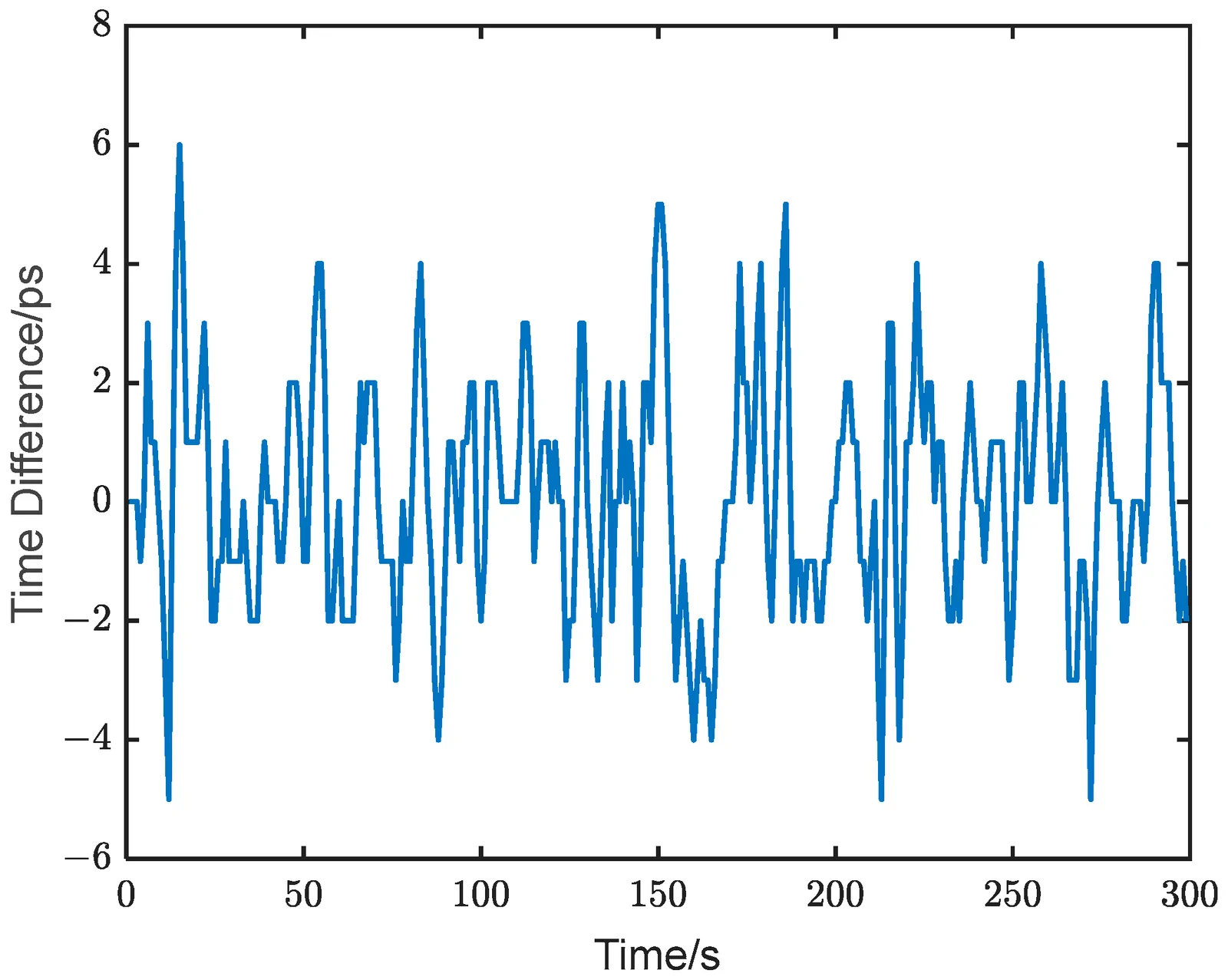
Time difference measurement.

This section details the hardware validation. As illustrated in the Figure 13, the post-synchronization clock offset measurement over a 300 s interval yields a standard deviation of 1.97 ps. The results verify that the proposed scheme exhibits competent performance regarding time synchronization within the constraints of this experimental setup.

## 4. Discussion

### 4.1. Characteristics of Synthesizing the Stability of Two Clocks by Steering

The observations derived from Figure 7, which summarizes comparative experiments of the two steering schemes under a 4-s control interval (the configuration where both methods reach their optimal capacity for fully exploiting the inherent stability merits of the two referenced clocks), provide critical mechanistic insights into the behavioral discrepancies between the investigated controllers. The presented performance trajectory of the proposed method, which exhibits full consistency with the stability profile of the free-running OCXO for averaging times τ < 5 s and that of the free-running AHM for τ > 200 s, directly validates its inherent capability to preserve the uncompromised short-term stability of the local OCXO while seamlessly tracing the superior long-term stability of the AHM reference, with the steered OCXO sustaining a plateaued and undegraded stability profile across the intermediate 5 s < τ < 200 s range. For the LQG control cohort, the statistically non-significant performance differences (*p* = 0.43 for the cross-group comparison between Trial 1/Trial 3 and the proposed method; *p* = 0.44 for the cross-group comparison between Trial 4 and the proposed method) indicate that the parameter sets of Trial 1 and Trial 3 deliver marginally better short-term stability but deteriorated long-term stability relative to the proposed approach, whereas Trial 4 attains comparable long-term stability at the cost of compromised short-term performance. These systematic performance contrasts collectively corroborate that the proposed framework yields more favorable overall effectiveness than conventional LQG control in the targeted application scenario for complementary fusion of the distinct stability characteristics from heterogeneous clock sources, offering an optimized paradigm for high-precision single-satellite time reference generation.

### 4.2. Noise, Parameters and Steering Performance

To study how parameter selection affects the performance steering under noise, it is necessary to review the closed-loop error equations of the Kalman filter with controller. As for LQG control [9], since $H=\left[\begin{array}{ccc} 1 & 0 & 0 \end{array}\right]$, the Kalman gain could be rewritten as:

(31) $$K(t)=\frac{1}{C^{-}(t{)}_{1,1}+R}\left(\begin{array}{c} C^{-}(t{)}_{1,1}\\ C^{-}(t{)}_{2,1}\\ C^{-}(t{)}_{3,1} \end{array}\right)$$

Then, the state estimate with the controller could be rewritten as:

(32) $$\hat{x}\left(t\right)=\phi \left(\delta \right)\hat{x}\left(t-\delta \right)+Bu_{LQG}\left(t-\delta \right)+\frac{x_{1}\left(t\right)+r\left(t\right)-{\hat{x}}_{1}^{-}\left(t\right)}{C^{-}(t{)}_{1,1}+R}\left(\begin{array}{c} C^{-}(t{)}_{1,1}\\ C^{-}(t{)}_{2,1}\\ C^{-}(t{)}_{3,1} \end{array}\right)$$

where $B={\left(\begin{array}{ccc} \delta & 1 & 0 \end{array}\right)}^{T}$. The frequency state is selected from Equation (32):

(33) $${\hat{x}}_{2}\left(t\right)={\hat{x}}_{2}\left(t-\delta \right)+\delta {\hat{x}}_{3}\left(t-\delta \right)-{\hat{G}}_{0}\hat{x}\left(t-\delta \right)+\frac{x_{1}\left(t\right)+r\left(t\right)-{\hat{x}}_{1}^{-}\left(t\right)}{C^{-}(t{)}_{1,1}+R}C^{-}(t{)}_{2,1}$$

where ${\hat{G}}_{0}=\left[\begin{array}{cc} {{\hat{G}}_{0}}_{1} & {{\hat{G}}_{0}}_{2} \end{array}\right]$, and the difference in the frequency estimates during a control interval could be expressed as:

(34) $${\hat{x}}_{2}\left(t\right)-{\hat{x}}_{2}\left(t-\delta \right)=\delta {\hat{x}}_{3}\left(t-\delta \right)-{\hat{G}}_{0}\hat{x}\left(t-\delta \right)+\frac{x_{1}\left(t\right)+r\left(t\right)-{\hat{x}}_{1}^{-}\left(t\right)}{C^{-}(t{)}_{1,1}+R}C^{-}(t{)}_{2,1}$$

where ${\hat{x}}_{3}\left(t-\delta \right)$ denotes the frequency drift, which is not changed during the steering; $-{\hat{G}}_{0}\hat{x}\left(t-\delta \right)$ denotes the frequency steering value. The smaller the value of ${\hat{x}}_{2}\left(t\right)-{\hat{x}}_{2}\left(t-\delta \right)$ is, the better the frequency stability.

**Figure 14:**
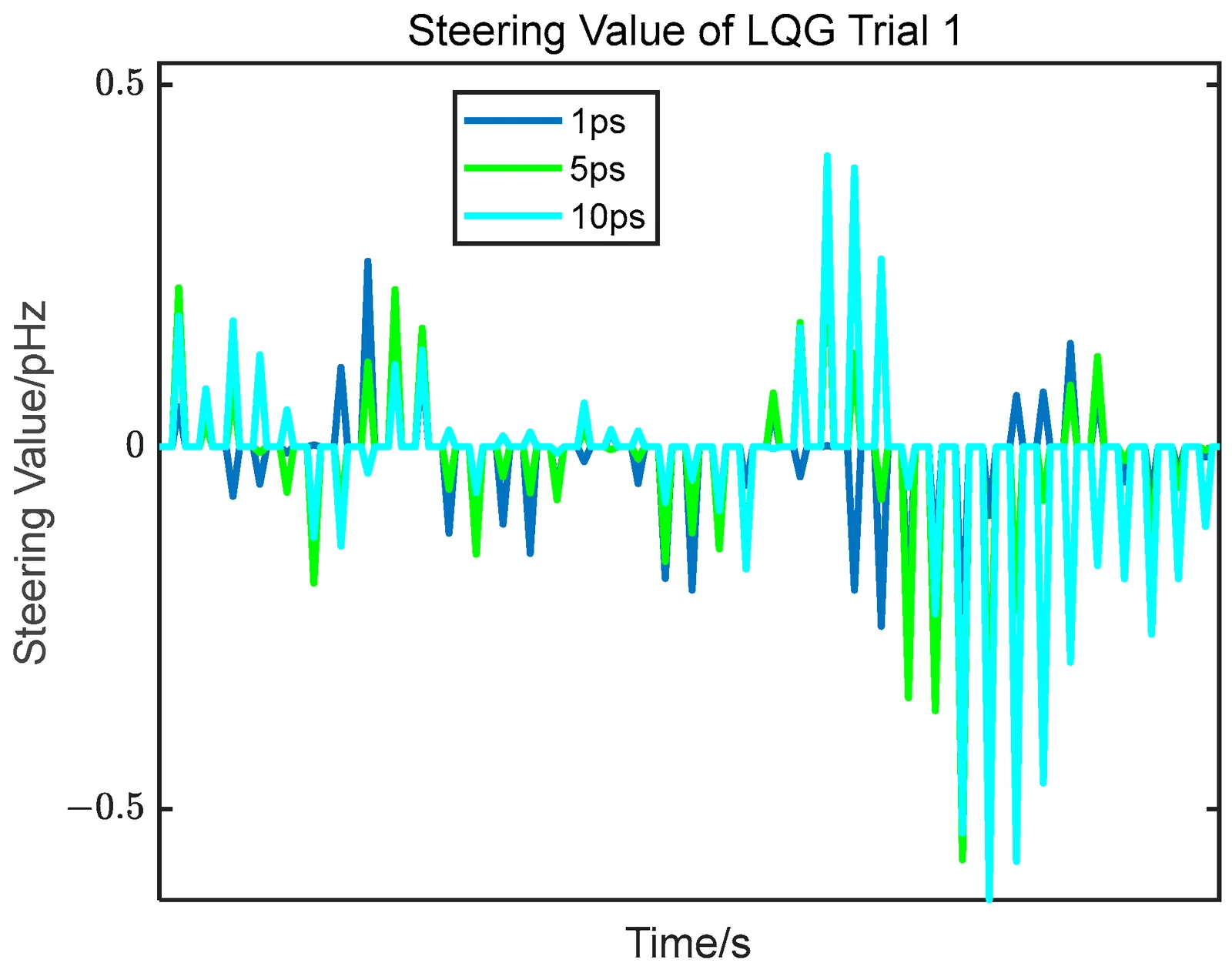
Some steering values of LQG Trial 1.

Notably, the core distinction between the proposed framework and conventional strategies lies in the explicit formulation of the steering quantity, as rigorously defined in Equations (15)–(20). A judicious parameter tuning paradigm enables substantial mitigation of noise-induced disturbances, thereby yielding superior frequency stability, as empirically verified by the performance of LQG Trial 1. Conversely, improperly configured parameters may inadvertently amplify the adverse effects of noise fluctuations, a phenomenon well illustrated by the outcomes of LQG Trial 4. As established in Section 4.1, elevated measurement noise introduces escalated estimation uncertainty, which in turn degrades the accuracy of frequency steering values and may even induce directional inversion of the steering output, as graphically depicted in Figure 14.

This underlying mechanism fundamentally accounts for the observed deterioration in the stability of the ultimately steered signal. For LQG Trial 1, the intrinsic low time synchronization accuracy renders its frequency stability nearly insensitive to the amplified measurement noise. As summarized in the phase MSE metrics visualized in Figure 10 for the proposed method, the datasets optimized via cost function $J_{1}$ yield phase MSE values of 119.93 (1 ps noise), 765.2 (5 ps noise) and 1094.2 (10 ps noise), while the counterparts deployed with cost function $J_{2}$ produce corresponding values of 83.2 (1 ps noise), 711.8 (5 ps noise) and 1018.1 (10 ps noise). Two robust conclusions can thus be distilled from the above results: First, the simultaneous achievement of optimal comprehensive frequency stability and maximum time synchronization accuracy is theoretically and practically unattainable. Second, for parameter configurations designed for high-precision time synchronization, amplified measurement noise predominantly impairs the short-term stability of the steered signal. Nevertheless, by carefully selecting the trade-off parameters to moderately relax the partial time synchronization accuracy, the noise interference can be dramatically suppressed.

**Figure 15:**
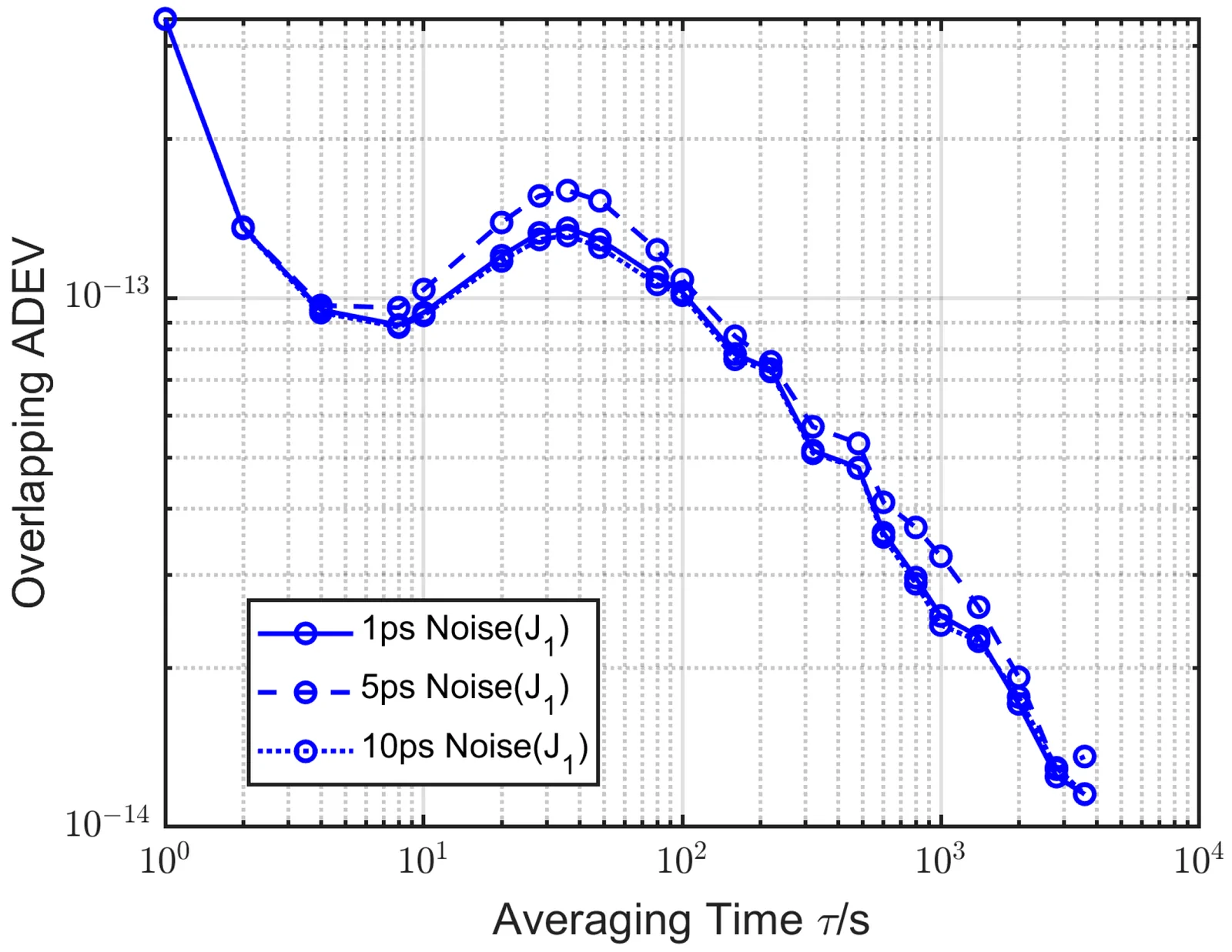
Overlapping ADEV of the results of proposed method (after searching optimal parameters for different noises) under different measurement noise.

Accordingly, adaptive parameter optimization targeted at varying noise regimes emerges as a pivotal practical challenge for the real-world deployment of single-satellite high-precision time reference generation systems. Notably, to the best of our knowledge, no published investigation has systematically addressed the parameter tuning of LQG frameworks for the specific scenario of atomic clock ensemble time scale generation for on-orbit satellite platforms. In contrast, the parameter calibration for our proposed methodology can be straightforwardly implemented via the explicit optimization framework elaborated in this work. On this basis, we further performed a targeted global optimization search to derive the optimal parameter subsets that are customized for distinct noise intensity levels, and the corresponding simulation-derived performance outcomes are visualized in Figure 15. Current research has not realized further optimization for the parameter searching process, and relevant targeted studies will be carried out in the future to effectively promote the overall searching efficiency.

## 5. Conclusions

This work proposes a novel clock steering scheme for high-precision time reference generation on single satellites, effectively disciplining OCXOs against AHM references. Beyond the comprehensive numerical simulations and comparisons with the classic LQG control framework presented earlier, we have conducted hardware experiments to further validate the proposed method. These experiments specifically confirm the scheme’s superior performance in terms of time synchronization.

However, this study is subject to certain limitations. As indicated by our analysis, steering methods with higher intrinsic time synchronization accuracy—such as the proposed scheme—are more susceptible to performance degradation induced by elevated measurement noise, which predominantly affects short- and medium-term stability. While our proposed parameter optimization strategy can mitigate these losses, it relies on specific tuning that may vary across different hardware setups.

Future research will focus on extensive experimental validation under more complex, dynamic orbital environments. We aim to verify the robustness of the proposed parameter tuning strategy and further assess the long-term reliability of the hardware implementation in real-world space scenarios.

## Figures and Tables

**Table 1:** Other studies with similar goals.

Source	Methodology	Scenario
Proposed Method	VBKF+PID	Dual-Clock System
Meng [2]	Third-Order FLL	LEO Receivers
Wang [20]	FDCI+LQG	LEO Satellites
Van Buren [21]	Oscillator+GPS Receiver	LEO Satellites

**Table 2:** Diffusion coefficients for simulation.

	$q_{1}$	$q_{2}$	$q_{3}$
OCXO	$7.6\times 10^{-28}$	$1\times 10^{-27}$	$6.1\times 10^{-51}$
AHM	$6.31\times 10^{-25}$	$1.1\times 10^{-35}$	$4.4\times 10^{-51}$

**Table 3:** Parameter settings for the LQG controllers.

Parameter	Value
$W_{Q}$ (Trial 1)	$\left(\begin{array}{cc} 10^{-4} & 0\\ 0 & 10^{-9} \end{array}\right)$
$W_{R}$ (Trial 1)	$1\times 10^{12}$
$W_{Q}$ (Trial 2)	$\left(\begin{array}{cc} 10^{-3} & 0\\ 0 & 10^{-3} \end{array}\right)$
$W_{R}$ (Trial 2)	$1\times 10^{12}$
$W_{Q}$ (Trial 3)	$\left(\begin{array}{cc} 10^{-4} & 0\\ 0 & 10^{9} \end{array}\right)$
$W_{R}$ (Trial 3)	$1\times 10^{12}$
$W_{Q}$ (Trial 4)	$\left(\begin{array}{cc} 10^{-4} & 0\\ 0 & 10^{-9} \end{array}\right)$
$W_{R}$ (Trial 4)	1

**Table 4:** Parameter settings for the PID controllers.

Parameter	Control Interval	Value
$K_{p}$	4 s	0.27
$K_{i}$		0.01
$K_{d}$		0.34
$K_{p}$	10 s	0.59
$K_{i}$		0.03
$K_{d}$		0.08
$K_{p}$	20 s	0.82
$K_{i}$		0.08
$K_{d}$		0.06
$K_{p}$	40 s	0.80
$K_{i}$		0.25
$K_{d}$		0.11

**Table 5:** *p*-value corresponding to the average time of two steering methods.

Control Interval	4 s	10 s	20 s	40 s
Proposed Method	0.14	0.20	0.24	0.42
LQG Trial 1	0.43	0.60	0.97	1.82
LQG Trial 4	0.44	0.48	0.66	1.13

**Table 6:** Phase MSE of different simulations (${ps}^{2}$).

Control Interval	4 s	10 s	20 s
$\mathrm{Proposed}\;\mathrm{Method}\;(J_{1}$)	71.4	1727.5	2192.4
$\mathrm{Proposed}\;\mathrm{Method}\;(J_{2}$)	38.6	616.3	1360.7
LQG Trial 1	5920.2	6028.6	6323.4
LQG Trial 4	15.8	39.7	86.9

**Table 7:** Statistical analysis of the experimental results.

	Mean ± STD (ps)	95% CI (ps)	*p*-Value
PM	−1.76 ± 13.39	[−1.87, −1.66]	*p* < 0.001
LQG Trial 4	−0.28 ± 15.89	[−0.41, −0.15]	-

Note: Data are presented as mean ± standard deviation (STD). 95% CI: 95% confidence interval. *p*-values were calculated using Student’s *t*-test to compare the proposed method against the baseline. *p* < 0.001 indicates statistical significance.

## Data Availability

The original contributions presented in this study are included in the article. Further inquiries can be directed to the corresponding author.

## Abbreviations

The following abbreviations are used in this manuscript:

OCXO	Oven-Controlled Crystal Oscillator
AHM	Active Hydrogen Maser
PID	Proportional-Integral-Derivative
LQG	Linear Quadratic Gaussian
GNSS	Global Navigation Satellite System
GPS	Global Position System
PFS	Phase, Frequency and Steers
KF	Kalman Filter
VBKF	Variational Bayesian Kalman Filter
